# Attribute Selection Impact on Linear and Nonlinear Regression Models for Crop Yield Prediction

**DOI:** 10.1155/2014/509429

**Published:** 2014-05-26

**Authors:** Alberto Gonzalez-Sanchez, Juan Frausto-Solis, Waldo Ojeda-Bustamante

**Affiliations:** ^1^IMTA, Boulevard Cuauhnáhuac 8532, Colonia Progreso, 62550 Jiutepec, MOR, Mexico; ^2^UPEMOR, Boulevard Cuauhnáhuac 566, Colonia Lomas del Texcal, 62550 Jiutepec, MOR, Mexico

## Abstract

Efficient cropping requires yield estimation for each involved crop, where data-driven models are commonly applied. In recent years, some data-driven modeling technique comparisons have been made, looking for the best model to yield prediction. However, attributes are usually selected based on expertise assessment or in dimensionality reduction algorithms. A fairer comparison should include the best subset of features for each regression technique; an evaluation including several crops is preferred. This paper evaluates the most common data-driven modeling techniques applied to yield prediction, using a complete method to define the best attribute subset for each model. Multiple linear regression, stepwise linear regression, M5′ regression trees, and artificial neural networks (ANN) were ranked. The models were built using real data of eight crops sowed in an irrigation module of Mexico. To validate the models, three accuracy metrics were used: the root relative square error (RRSE), relative mean absolute error (RMAE), and correlation factor (*R*). The results show that ANNs are more consistent in the best attribute subset composition between the learning and the training stages, obtaining the lowest average RRSE (86.04%), lowest average RMAE (8.75%), and the highest average correlation factor (0.63).

## 1. Introduction

Crop yield prediction (CYP) is important for agricultural planning and resource distribution decision making [1]. Regrettably, CYP is a difficult task because many variables are interrelated [2]. Yield is affected by human producer decisions or activities (such as irrigated water, land, and crop rotation) or incontrollable factors (such as weather). Commonly, cropping planners use the previous yield as an estimation of future yield. Nevertheless, crop yield varies spatially and temporally with a nonlinear behavior, introducing large deviations from one year to another [3]. Thus, more efficient methods for CYP have been developed, in which crop growth and data-driven models are the most popular. Crop growth models, using site-specified experimental data, regional calibration, and plot level observations, are recognized as robust and efficient models. However, they are available only for some crops, with development time and cost being extremely large [3]. On the other hand, data-driven models work with high-level information and are built empirically without a deep knowledge about physical mechanisms which produce the data. Previous works suggest that data-driven models have better adaptability for cropping planning than crop growth methods due to their friendly implementation and performance [4].

Data driven models are widely applied using classical statistics and data-mining methods. Statistical models use parametric structures tuned with sum-of-squares residuals, validated by hypotheses test and confidence intervals. Most of the statistical applications for CYP have been linear [3], obtaining a range from bad to moderate results. Data mining applies machine learning techniques and nonparametric structures, in which validation uses prediction accuracy. Machine learning (ML) obtains nonlinear models from massive datasets [5]. Most common ML techniques for CYP are regression trees [6] and neural networks [3, 4, 7]. Despite the high site dependency, neural networks are widely recognized as robust models, obtaining good results for CYP [7]. Comparisons between linear and nonlinear models for CYP show a small advantage in favor of nonlinear models [3, 7]. However, the attribute subset is usually the same for all the evaluated techniques. In practice, the explanatory attributes are selected from expertise assessment or previous publications, for instance, [3, 8]. However, the explanatory attributes may have a different impact on each technique, even using the same dataset [9]. A fairer comparison should include the best attribute subset for each technique, selected with some performance metrics [10]. Regrettably, only an exhaustive approach can guarantee the optimal subset for all regression techniques. Some CYP datasets have relatively few attributes and an exhaustive approach can be applied to model comparison purposes [8].

In this paper, a comparison between linear and nonlinear data-driven modeling techniques for CYP is presented. The best attribute subset for each technique is determined by measuring the predictive accuracy of each model subset. To obtain the optimal subset, a recursive algorithm finds all the feature combinations, building a regression model of each subset. The models are built using most samples of training datasets, leaving the more recent to measure the performance. The best subset for each technique is tested with samples representing future information which had not been included in the training stage. The most common techniques for CYP were compared: multiple linear regression, stepwise linear regression, M5′ regression trees, and perceptron multilayer neural networks. Results per technique are compared against those obtained using the optimal attribute combination derived from the test dataset. The potential attributes considered for this work were irrigation water depth (mm), accumulated rainfall (mm), solar radiation (MJ/m^2^), maximum and minimum temperatures (°C), relative humidity (%), and the farm location. To build the models, historical data of eight crops were obtained from one irrigation module located in Mexico. Results show the best CYP technique, the most influential attributes for each model, and the fact that an exhaustive approach on the training dataset does not guarantee optimality on testing dataset.

This paper is organized as follows.  Section 2 describes data sources, data-driven techniques, accuracy metrics, and the recursive algorithm used to build and test the models.  Section 3 shows the experimental results and discussion. Finally, Section 4 presents the conclusions about realized work.

## 2. Materials and Methods

### 2.1. Data Description

This paper uses data obtained from the irrigation district 075 (Santa Rosa III-1 module) in Sinaloa, Mexico (one of the largest and most productive districts in the country). Two data sources from the year 1999 to 2007 were collected: (a) agricultural production data and (b) weather information data. The former included attributes regarding sowed areas, crop types, quantity of irrigated water, starting and ending sowing dates, and crop yield. Such data were obtained from Spriter-GIS system [11]. The second data source includes climatological variables measures such as rainfall, solar radiation, and temperatures. Weather data were collected from the National Meteorological Service (SMN) stations located in the module vicinity. The CRISP-DM [5] methodology was applied to clean, homogenize, and integrate both data sources into one single database, obtaining eight crop representative datasets. Eight potential attributes (Table 1) were selected based on previous CYP works [12] and the data availability. Such attributes are referred to as* potential* because this work uses a complete algorithm to find the best attribute subset for each regression technique. Thus, the final subset of attributes depend on the algorithm execution. Average of weather attributes (solar radiation, temperatures, and humidity) was estimated with the last three crop growing stages, the most influential in the crop development.

The crop datasets are described in Table 2. To simplify future references of these datasets, an ID is assigned to each one (which is shown in the first column). Table 2 describes the quantity of records and periods of time used for the training and testing stages. In order to maintain realistic conditions, the last year of available data was reserved for testing.

### 2.2. Data-Driven Modeling Techniques

The most common data-driven techniques applied to CYP were selected for this work: multiple and stepwise linear regression [3, 7], M5′ regression trees [2, 8, 13], and artificial neural networks [1, 3, 7, 12].

#### 2.2.1. Multiple and Stepwise Linear Regression

Multiple linear regression (MLR) is a popular technique which can be applied to predict a dependent variable *Y*_*i*_, using a set of independent variables *X*_*ij*_. MLR model is described by [14]

(1) $$\begin{array}{c} Y_{i}=\overset{k}{\underset{j=1}{\sum }}B_{j}X_{ij}+\epsilon_{i}, \end{array}$$

where *k* is the number of independent variables, *B*_*j*_ is a regression coefficient, *X*_*ij*_ is the *j* value for the observation *i*, and *ϵ*_*i*_ is the residual error. If *X* ^*T*^ *X* is a nonsingular matrix, an approximation for $B\left(\overset{-}{\beta }\right)$ can be obtained by $\overset{-}{\beta }={\left(X^{T}X\right)}^{-1}X^{T}Y$. Then (1) can be rewritten as $Y=X\overset{-}{\beta }+\epsilon$.

Stepwise linear regression (SLR) works with the same principle. However, SLR performs a semiautomated selection on independent variables to maximize the model's prediction efficiency. Linear regression is performed by adding or removing independent variables on each iteration. Initially, the variable with the highest correlation (*R*-squared) measured with respect to the dependent variable is included. Then, the remaining independent variable with the highest correlation with respect to the dependent variable is selected. This iterative process is repeated while the addition of a remaining independent variable increases *R*-squared with a significant quantity. We use the SLR implementation in SPSS [15], which combines forward selection and backward elimination [16]. At each step, the best remaining variable is added according to a significance criterion *α* of five percent; then the entire set of variables is reviewed to decide whether a single variable is removed using an *α* of ten percent.

#### 2.2.2. Regression Trees

A regression tree (RT) is based on a decision tree, a classifier expressed as a recursive partition of the samples' space [17]. A tree is formed by nodes, in which the first is named the root node (without incoming edges). All the other nodes have exactly one incoming edge. A node with outgoing edges is called a test node and a node without outgoing edges is called a leaf node. Each internal node in the tree splits the samples' space into two or more subspaces based on conditions of the input attributes values. In the case of numerical attributes the condition refers to a range of values. Each leaf is assigned to one class representing the most appropriate target value. Samples are classified by navigating them from the root of the tree down to a leaf, according to the outcome of the tests along the path. For regression trees, the class at the leaf nodes assigns a numerical value to the tested sample which corresponds to the value predicted by the regression model. The most common algorithms to build RTs are CART, M5, and M5′ [17]. This work uses M5′ algorithm implemented by Weka [17]; the standard deviation reduction (SDR) is applied as a measure of impurity on continuous attributes. The parameters to build an RT with a minimum of two samples by node, pruned and smoothed, were selected.

#### 2.2.3. Artificial Neural Networks

From a structural point of view, an artificial neural network (ANN) is a collection of simple processing units linked via directed and weighted interconnections. Each processing unit receives a number of inputs from the outside or other processing units. Each input is calibrated based on the weights of their interconnections. Once calibrated, inputs are combined and transmitted to other processing units via the appropriate interconnections. The units are organized by layers, hiding the intermediate layers to the user. This process is represented by a nonadditive and nonlinear function that maps the set of inputs to a set of outputs [17]. The training stage is an iterative process performed to pound connections and it is guided for error measure. There are many ANN topologies and training algorithms. This work uses the most popular topologies and learning algorithm combinations: multilayer perceptron (MLP) and backpropagation algorithm [17]. MLP network has been a popular choice for CYP [1, 3]. Backpropagation algorithm minimizes the error function using the gradient descent method. The combination of weights obtained is a solution of the learning problem. Since this method requires computation of the gradient of the error function at each iteration step, the continuity and differentiability of this function should be verified. In addition, an activation function is required where the sigmoidal function (1/(1 + *e* ^−*cx*^)) is commonly used [17]. In this work, a topology with three layers and 10 neurons on a single hidden layer was used; this topology was applied in other works [4]. The most recommended parameters were applied such as the weight decay and numeric attribute normalization [3]. Training epochs, learning rate, and the momentum were established by experimentation, being 1000, 0.3, and 0.01, respectively. Quantity of neurons at the input layer depends on number of attributes (see Section 2.5), while the output layer has only a neuron (CYP estimation).

### 2.3. Accuracy Metrics

We use three of the most common metrics of regression models [5]: the root relative square error (RRSE), correlation factor (*R*), and the relative mean absolute error (RMAE). RRSE compares the model prediction against the mean, which is frequently used to supply the crop yield value. An RRSE less than 100% indicates a prediction that is better than the average value. Correlation factor (*R*) measures the linear relationship between regression model predictions and the real values. Mean absolute error (MAE) is the average of estimation differences (in physical units). This metric is expressed as a percentage relative to the mean yield, being called RMAE instead of MAE. Equation (2) shows how these metrics are calculated, where *y* is the real yield value, $\hat{y}$ represents the yield estimation, *i*  is the number of sample, $\overset{-}{y}$ is the average of the real yield values, and $\overset{-}{\hat{y}}$ is the average of predictions:

(2) $$\begin{array}{c} \text{RRSE}\left(\%\right)=\sqrt{\frac{\sum_{i=1}^{n}{\left(y_{i}-{\hat{y}}_{i}\right)}^{2}}{\sum_{i=1}^{n}{\left(y_{i}-\bar{y}\right)}^{2}}}\times 100,\\ R=\frac{\sum_{i=1}^{n}\left(y_{i}-\overset{-}{y}\right)\left({\hat{y}}_{i}-\overset{-}{\hat{y}}\right)}{\sqrt{\sum_{i=1}^{n}{\left(y_{i}-\overset{-}{y}\right)}^{2}}\sqrt{\sum_{i=1}^{n}{\left({\hat{y}}_{i}-\overset{-}{\hat{y}}\right)}^{2}}},\\ \text{RMAE}\left(\%\right)=\left(\frac{\sum_{i=1}^{n}\left|y_{i}-\hat{y_{i}}\right|}{\left(n\right)\left(\overset{-}{y}\right)}\right)\times 100. \end{array}$$

Many CYP works use root mean squared error (RMSE) as accuracy metric. RMSE measures the difference between real and estimations values, exaggerating the presence of outliers [5]. We use RRSE instead of RMSE because the former applies the average value as common reference point, being easy to understand by people unaccustomed to physical crop yield dimensions.

### 2.4. Method to Find the Best Attribute Subset

A combinatorial procedure to perform a complete enumeration of all the subsets {**x**_1_, **x**_2_, **x**_3_,…, **x**_**m**_} is presented in this paper. The procedure starts with a potential set of attributes *A* = {*a*_1_, *a*_2_,…, *a*_*n*_}, such that each **x**_1_ is a subset of *A*. Each **x**_**k**_ subset is evaluated using the training dataset, which is divided in two datasets. The majority of samples are used to build the models, while the most recent ones are applied for performance measurement. In CYP context, if the [**a**, **b**] year range of historical data is available for training, the [**a**, **b** − 1] range is really used for training, and data from year **b** is reserved for validation. The model's validation is made using the metrics described in Section 2.3. Each validation result and the related attribute subset are registered in a sorted list according to these metrics. Ties are solved in the following order: RRSE (lower), *R* (higher), and RMAE (lower). At the end of the process, the subset at the top is taken as the best.  Algorithm 1 shows the algorithm of the optimal attribute search process.

The function* evalModel(model, validSamples)* of Algorithm 1 evaluates the argument* model* with samples taken from the* validSamples* dataset. This function uses the percentage-split validation scheme approach [5]. We tried other validation schemes as well, such as training and validating the models with the entire training dataset and cross-validation (CV). The former provided very poor results to predict the yield of future samples. On the other hand, CV (considered a robust validation scheme) was difficult to apply because (1), for *k* subsets required for CV, *k* − 1 models should be stored, and (2) the computational cost of the entire process is increased *k* − 1 times for each evaluation [3], being not computationally tractable in practical applications.

### 2.5. Distance to the Optimal Attribute Subset (OAS)

Algorithm 1 can be applied to both the learning and testing stages. When this algorithm is applied to the former, a ranking of attribute combinations is obtained, placing the best attribute combination at the top. This subset is named the learning attribute subset (LAS). In testing stage, the algorithm is applied to the union of the training and the testing datasets, obtaining a rank of attribute subsets. In this last case, the subset at the rank's top is named the optimal attribute subset (OAS). Evidently, this last rank cannot be available in practice, because testing dataset represents unseen samples from the future. However, the rank of attribute combinations that originated the OAS can be used to define a new performance metric, which should be used only for evaluation purposes. Let *x* be an attribute subset and *D* the number of combinations that separates the OAS results from the *x* subset results. Then *D* can be used as a performance measure of *x*. We called measure *D* the “distance to the optimal attribute subset.”

## 3. Results and Discussion

Experimental results are presented in the next three sections.  Section 3.1 shows the metric measures obtained in testing dataset with the OAS.  Section 3.2 shows the metric measures using the potential attributes.  Section 3.3 describes the results using the LAS on testing dataset.

### 3.1. Metric Measures Using the OAS on Testing Dataset

The OAS for the testing dataset to each crop technique was obtained with the algorithm of Section 2.4.  Table 3 shows every metric obtained per technique (RRSE, *R*, and RMAE). RRSE shows that all techniques achieve better predictions than the average. For the potato crop datasets (PA06 and PA07), MLR obtains only slightly better results than the average (RRSE of 95%). In general, nonlinear techniques show some improvements over MLR, introducing small RRSE measures and *R* values near to 0.7.

Tables 4(a), 4(b), 4(c), and 4(d) show the OAS composition found for each regression technique. The attributes in OAS are shown in shaded cells. Evidently, OAS is the same for SLR and MLR (Tables 4(a) and 4(b), resp.). Attributes selected are grouped in Table 5, which shows the quantity of times that a particular attribute is included in the OAS for each crop dataset. The average column in Table 5 indicates that the IWD, RH, SGR, and MINT attributes appear in more than half of optimal crop yield models, mostly independent of the regression technique. Besides, IWD (irrigation water depth) was the attribute most selected by all techniques.

**Figure 1:**
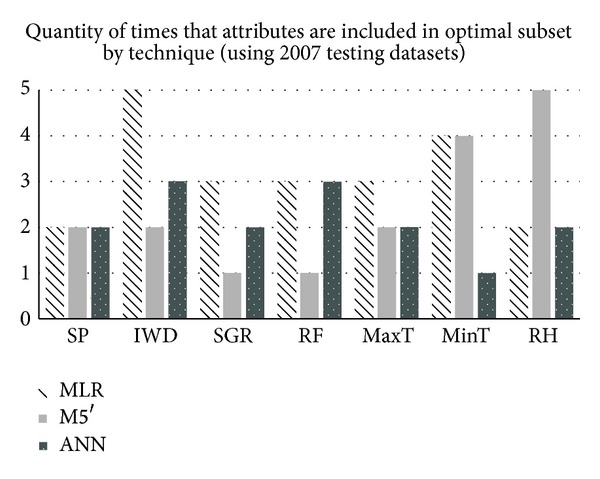
Quantity of occurrences of each attribute in the OAS for each technique (only crop datasets with 2007 testing data).

Because attributes selected can be influenced by temporal elements, Figure 1 shows the results obtained only with the five crop testing datasets of year 2007. Attributes most frequently selected by MLR technique were MinT and IWD, with the latter always included in the OAS. Attributes most frequently selected by M5′ were MinT and RH, with the latter always included in the OAS. On the other hand, attributes selected by ANN technique were IWD and RF. Unlike other techniques, ANN did not always select a specific attribute.

### 3.2. Metric Measures Using All the Potential Attributes

Table 6 shows the RRSE, *R*, and RMAE measures using all the potential attributes as explanatory variables. RRSE indicates that only two of the eight crop models per technique obtain better predictions than the mean yield value. MLR has three models with good predictions. However, PA06 model shows an *R* value of 0.07, indicating a very low linear relationship between the prediction and the real yield. The models for the PJ01 dataset are the most consistent, showing good results with every technique and a small improvement with nonlinear models. For every technique, the set of RRSEs lower than one hundred percent was averaged; in the case of Table 6, the figures obtained were 94.41, 74.34, and 75.79 for MLR, M5′, and ANN, respectively. The averages with the entire set of RRSEs were also calculated and shown in the row named* Average (all)* of Table 6. We decided to average the RMAEs with an RRSE lower than one hundred percent and an *R* factor close to one and greater than a threshold value, set as 0.6 in this work. As is well known, a good prediction model should have a low RRSE and an *R* value close to 1. Therefore, for all the potential attributes and when only RRSE and *R* are considered, we can observe that M5′ is the best technique. Averaging the RMAEs that accomplish this criterion (RRSE < 100% and an *R* > 0.6), the best techniques were again M5′ and ANN.

The distance *D* to the optimal attribute subset (described in Section 2.5) provides an idea of how far are the OAS results to those obtained with all the potential attributes.  Table 7 shows *D* values for the evaluated techniques, which indicates that very few models are close to the optimal results using all the potential attributes. Considering all the 256 possible combinations, most of the obtained results with all the attributes are located beyond the middle of the rank of combinations.

### 3.3. Metric Measures Using the LAS on Testing Dataset

The LAS for each crop technique was obtained during the learning stage using only data from each training dataset. The models built with the LAS were applied to predict the yield of samples on testing dataset. In addition, SLR has its own attribute selection mechanism and is included in this section. RRSE and *R* measures in Table 8 show the obtained results. Our attribute selection algorithm (Section 2.4) improves the MLR, M5′, and ANN models performance, increasing the number of CYP models with RRSE measures lower than 100% (MLR obtained five models, while M5′ and ANN obtained six models each). SLR has a poor performance, with only one model with an RRSE measure lower than 100%, and an average error even higher than MLR using all the potential attributes (Table 9). RRSE of nonlinear techniques shows small improvements with respect to MLR. In addition, *R* measures of MLR are higher than those obtained by M5′ and inferior to those obtained with ANN. Average *R* values for the models with RRSE lower than 100% are greater than 0.5. Only nonlinear techniques obtained an average RRSE value lower than 100%. Among these, ANN obtained better results, with the lowest RRSE, the highest *R*, and the lowest RMAE measures.

Table 9 shows that MLR, M5′, and ANN model errors built with the LAS decrease considerably when they are compared against the use of all the potential attributes. Average RMAE values decreased as follows (in %): (a) for MLR, from 17.54 to 13.59; (b) for M5′, from 19.49 to 11.93; and (c) for ANN, from 18.23 to 12.89. The average RMAE measure for SLR was scored worse than MLR using all the potential attributes, obtaining an RMAE of 18.65%.  Table 10 shows the distances *D* of the error measure obtained from the LAS to the OAS results. ANN is the regression technique with more CYP models closer to the optimal. MLR and M5′ present similar average results. SLR was too far to optimal combination.

**Figure 2:**
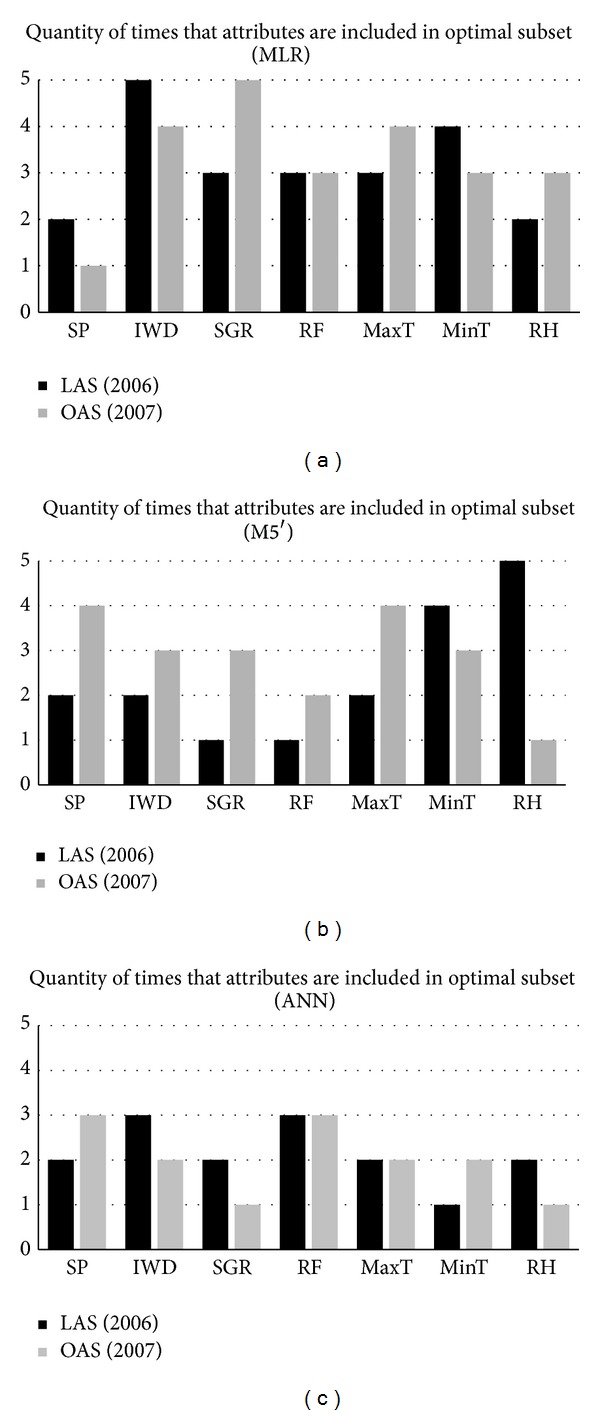
Quantity of occurrences of each attribute on the LAS and OAS for each technique (only crop datasets with 2007 testing data).

Tables 4(a), 4(b), 4(c), and 4(d) show the attributes contained in each LAS grouped per technique. These attributes are identified with a √ symbol. As it can be seen, LAS and OAS differ, showing that the best attribute subset can vary from one year to another. Nevertheless, such behavior is different for each technique. Let us illustrate the situation with Figures 2(a), 2(b), and 2(c), which show the frequency when an attribute appears in the LAS and OAS for the MLR, M5′, and ANN regression techniques (resp.). To avoid mixing results from different years, these charts only include attribute subsets obtained from the crop datasets with 2007 testing data (CBP02, CBA03, CBM04, PA06, and PA07). Figures 2(a) and 2(c) show that OAS and LAS are similar for MLR and ANN techniques. On the other hand, LAS and OAS obtained with M5′ technique are completely different in almost all cases (Figure 2(b)). As a result, ANN is the most consistent technique since its best attribute subsets scarcely varied from year to year (Figure 2(c)).

We applied the *R* measure to the attributes in LAS that intersects the OAS and the RRSE. This allows us to distinguish the error caused by including the relevant attributes in the model and the errors due to the regression technique predictive ability. This measure is shown in column two of Table 11. We also applied the *R* metric to the attributes excluded from LAS and those selected in OAS with the RRSE. This *R* correlation is shown in the third column of Table 11. These measures are included for SLR, MLR, and M5′ techniques. From column two in this table we observe that variables included in LAS and OAS have a strong effect over the error metric for SLR and M5′, while the effect of these variables scarcely affects the ANN performance. The third column of Table 11 indicates that the attributes excluded from OAS and included in LAS have a very small effect in M5′ and MLR, while a moderate impact over ANN and SLR can be observed. As a consequence, a biggest error among all these techniques can be expected from SLR.

## 4. Conclusions

This paper presents a comparison among several methods (linear and nonlinear) for crop yield prediction. The comparison is made using the best attribute subset found in the training dataset for each method, which was detected using a complete algorithm and the percentage-split validation scheme. The algorithm uses the oldest samples in training datasets to build the models, leaving the most recent to search the optimal attribute subset. The best attribute subset performance is measured with testing datasets composed of unseen samples from the future. The comparison covered eight crop datasets. The most common data-driven techniques for crop yield prediction were evaluated: stepwise linear regression, multiple linear regression, regression trees, and neural networks. The experimentation shows that our attribute selection using a complete method substantially improves the performance of all the evaluated techniques. ANN and M5′ obtained the best prediction, and, between them, the former achieved the lower RRSE, the higher R correlation, and the lower RMAE value. With respect to the optimal attribute subset composition, MLR and ANN techniques show small differences between the best attribute subset in learning stage and the optimal attribute subset for the testing stage, while M5′ shows the largest differences. The optimal attribute composition was different for all the evaluated techniques, which reinforces the hypothesis that using the same attributes subset for all the techniques is unfair. Nevertheless, none of the techniques was able to obtain the optimum subset with the training data for all the eight crops. The best technique was ANN, which achieved three attribute subsets equal to the optimal, and the other two subsets were very close to it. Thus, an attribute subset that can be used permanently in all the years for all the crops is difficult to select.

Results obtained from machine-learning methods cannot be directly applied to a different set of crop databases, due their high data dependency. However, the procedure presented in this paper can be extended for a larger number of techniques and crop datasets. A future research focused on finding the best minimal subset of attributes which provide a good yield of predictions on other irrigation zones should be done.

## Figures and Tables

**Algorithm 1:**
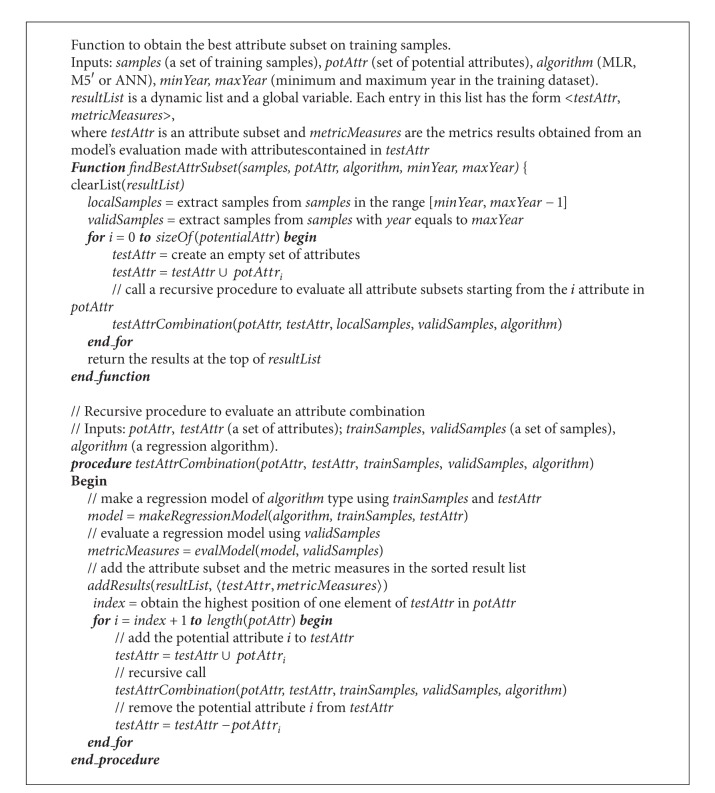
Recursive algorithm to perform the optimal attribute subset search.

**Table 1:** Potential attributes in crop datasets.

Attribute code name	Attribute description
SP	Section (farm location where crop was sowed)
IWD	Irrigation water depth applied (mm)
SGR	Solar radiation (M-Joules/m^2^)
RF	Rainfall (mm)
Max*T*	Maximal temperature (°C)
Min*T*	Minimal temperature (°C)
RH	Relative humidity in leafs (%)

**Table 2:** Testing and training samples distribution per crop dataset.

Dataset ID	Crop species	Cultivar	Training period	Training samples	Testing period	Testing samples
PJ01	Pepper (*Capsicum annuum*)	Jalapeno	1999–2005	116	2006	18
CBP02	Common bean (*Phaseolus vulgaris*)	Peruano	1999–2006	361	2007	9
CBA03	Common bean (*Phaseolus vulgaris*)	Azufrado	1999–2006	120	2007	21
CBM04	Common bean (*Phaseolus vulgaris*)	Mayocoba	1999–2006	332	2007	27
CP05	Corn (*Zea mays*)	Pioneer 30G54	2000–2005	179	2006	19
PA06	Potato (*Solanum tuberosum*)	Alpha	1999–2006	1749	2007	116
PA07	Potato (*Solanum tuberosum*)	Atlantic	1999–2006	1062	2007	92
TS08	Tomato (*Lycopersicon esculentum* Mill.)	Saladette	1999–2005	182	2006	15

**Table 3:** RRSE, *R*, and RMAE measures using the OAS on testing dataset.

Crop dataset	RRSE (%)			*R*			RMAE (%)		
	MLR	M5′	ANN	MLR	M5′	ANN	MLR	M5′	ANN
PJ01	50.69	29.29	49.62	0.87	0.96	0.88	8.63	4.56	8.27
CBP02	52.14	58.85	58.05	0.67	0.68	0.67	5.67	6.40	6.41
CBA03	63.40	38.66	38.66	0.94	0.93	0.93	4.72	3.62	3.62
CBM04	70.53	71.20	75.04	0.69	0.59	0.58	1.30	1.59	1.58
CP05	87.83	83.52	87.59	0.72	0.65	0.70	8.13	6.39	8.46
PA06	95.28	74.02	86.16	−0.13	0.63	0.54	25.58	20.05	23.13
PA07	95.84	88.14	91.24	0.60	0.51	0.45	17.78	16.42	17.40
TS08	86.59	82.40	74.87	0.69	0.64	0.73	11.08	13.46	14.57

Average	75.29	65.76	70.15	0.63	0.70	0.72	10.36	9.06	10.43

**Table:** (a) SLR

Crop dataset	Attributes						
	SP	IWD	SGR	RF	Max*T*	Min*T*	RH
PJ01	***√***	∗	***√*∗**	***√***			∗
CBP02		∗		***√*∗**	***√*∗**	∗	***√*∗**
CBA03	***√*∗**	∗				***√*∗**	
CBM04	***√*∗**	∗	∗	***√***			
CP05	∗	***√*∗**	***√*∗**	∗		***√*∗**	∗
PA06	***√***	***√*∗**	∗	***√*∗**	∗	***√*∗**	∗
PA07		***√*∗**	***√*∗**	∗	***√*∗**	∗	
TS08	***√*∗**	∗	∗	***√*∗**	∗		∗

Count (OAS)	4	8	6	5	4	5	6

Count (LAS)	5	3	3	5	2	3	1

**Table:** (b) MLR

Crop dataset	Attributes						
	SP	IWD	SGR	RF	Max*T*	Min*T*	RH
PJ01	√	***√*∗**	∗	***√***	***√***	***√***	∗
CBP02		***√*∗**	***√***	***√*∗**	***√*∗**	***√*∗**	***√*∗**
CBA03	***√*∗**	***√*∗**	***√***	***√***	***√***	***√*∗**	***√***
CBM04	∗	∗	***√*∗**	***√***	***√***		
CP05	***√*∗**	***√*∗**	***√*∗**	∗		***√*∗**	∗
PA06		***√*∗**	***√*∗**	∗	∗	***√*∗**	***√*∗**
PA07		***√*∗**	***√*∗**	∗	***√*∗**	∗	
TS08	***√*∗**	∗	***√*∗**	∗	***√*∗**		∗

Count (OAS)	4	8	6	5	4	5	6

Count (LAS)	4	6	7	4	6	5	3

**Table:** (c) M5′

Crop dataset	Attributes						
	SP	IWD	SGR	RF	Max*T*	Min*T*	RH
PJ01	***√*∗**	***√*∗**	***√***		***√*∗**	***√***	***√*∗**
CBP02	***√*∗**		***√*∗**	***√***	***√*∗**	∗	***√*∗**
CBA03	***√***		***√***		***√***	***√*∗**	∗
CBM04	***√***	***√***	***√***	∗	***√***		∗
CP05	***√*∗**	∗	***√*∗**				
PA06	***√***	***√*∗**		***√***	∗	***√*∗**	∗
PA07	∗	***√*∗**			***√***	***√*∗**	∗
TS08	***√*∗**	∗	∗	∗	***√***	***√***	

Count (OAS)	5	5	3	2	3	4	6

Count	7	4	5	2	6	5	2

**Table:** (d) ANN

Crop dataset	Attributes						
	SP	IWD	SGR	RF	Max*T*	Min*T*	RH
PJ01	***√***	∗	***√*∗**	***√***	∗	∗	∗
CBP02				***√*∗**	***√*∗**		***√*∗**
CBA03	***√*∗**	∗				***√*∗**	
CBM04	***√***		∗	***√*∗**			
CP05		***√*∗**	***√*∗**			***√*∗**	
PA06	***√*∗**	***√*∗**		***√*∗**		***√***	∗
PA07		***√*∗**	***√*∗**		***√***		
TS08	***√*∗**		∗	***√***			∗

Count (OAS)	3	5	5	3	2	3	4

Count (LAS)	5	3	3	5	2	3	1

**Table 5:** Quantity of crop yield models where attributes appear as optimal.

Attribute	Regression technique			Average
	MLR	M5′	ANN	
SP	4	5	3	4.00
IWD	8	5	5	6.00
SGR	6	3	5	4.67
RF	5	2	3	3.33
Max*T*	4	3	3	3.33
Min*T*	5	5	3	4.33
RH	5	6	4	5.00

**Table 6:** RRSE, *R*, and RMAE measures using all the potential attributes.

Crop dataset	RRSE (%)			*R*			RMAE (%)		
	MLR	M5′	ANN	MLR	M5′	ANN	MLR	M5′	ANN
PJ01	**85.36**	**48.83**	**65.51**	**0.89**	**0.9**	**0.92**	**14.21**	**6.99**	**9.28**
CBP02	**99.85**	**99.85**	124.23	**0.63**	**0.63**	0.64	**10.25**	**10.25**	13.21
CBA03	136.96	156.29	**86.07**	0.76	0.77	**0.59**	14.99	15.33	**7.83**
CBM04	470.62	262.08	350.32	−0.66	−0.66	−0.68	11.2	6.54	8.05
CP05	102.68	362.5	123.61	0.36	0.08	0.54	10.12	32.25	11.75
PA06	**98.02**	102.87	110.24	**0.07**	0.15	0.19	**26.02**	27.56	27.93
PA07	110.86	165.41	113.18	−0.03	−0.13	−0.18	20.67	37.07	24.23
TS08	166.86	100.56	146.6	0.45	0.28	0.09	32.83	19.95	43.57

Average (RRSE < 100)	94.41	74.34	75.79	0.53	0.77	0.76	16.83	8.62	8.55
Count (<100)	3	2	2				3	2	2
Average (all)	158.9	162.3	139.97	0.31	0.25	0.26	17.54	19.49	18.23

**Table 7:** Distance from OAS error measures using the potential attribute set.

Crop dataset	Distance from optimal (combinations)		
	MLR	M5′
PJ01	38	14	18
CBP02	80	189	135
CBA03	111	118	32
CBM04	231	135	216
CP05	71	206	196
PA06	23	173	186
PA07	230	194	213
TS08	229	69	185

Average	127	137	148

**Table 8:** RRSE and *R* measures using the LAS on testing dataset.

Crop dataset	RRSE (%)				*R*			
	SLR	MLR	M5′	ANN	SLR	MLR	M5′	ANN
PJ01	203.86	**81.90**	**58.00**	**75.25**	0.87	**0.81**	**0.90**	**0.82**
CBP02	130.52	**55.05**	**74.67**	**58.05**	0.66	**0.52**	**0.73**	**0.67**
CBA03	98.76	136.96	112.45	**58.40**	0.64	0.76	−0.05	**0.98**
CBM04	479.43	306.29	**85.30**	**78.96**	−0.67	0.66	**0.27**	**0.61**
CP05	103.77	**91.06**	**94.50**	**87.59**	**0.50**	**0.69**	**0.52**	**0.70**
PA06	102.41	102.36	**85.96**	101.33	−0.42	−0.32	**0.55**	0.11
PA07	110.44	**97.49**	101.31	**91.24**	−0.06	**0.67**	0.09	**0.45**
TS08	112.85	**86.59**	**82.40**	137.48	0.42	**0.69**	**0.64**	0.69

Average (RRSE < 100)	98.76	82.41	80.14	74.92	0.50	0.67	0.60	0.71
Count (<100)	1	5	6	6				
Average (all)	167.76	119.71	86.82	86.04	0.24	0.56	0.46	0.63

**Table 9:** RMAE (%) measures using the LAS on testing dataset.

Crop	RMAE (%)			
	SLR	MLR	M5′	ANN
PJ01	40.61	**15.46**	**10.00**	**12.46**
CBP02	14.10	**5.16**	**8.41**	**6.41**
CBA03	10.11	14.99	12.35	**6.08**
CBM04	11.31	8.65	**1.72**	**1.72**
CP05	**10.11**	**8.81**	**8.04**	**8.46**
PA06	27.17	26.98	**23.10**	26.29
PA07	21.01	**17.61**	18.35	**17.40**
TS08	14.75	**11.08**	**13.46**	24.27

Average (RRSE < 100)	10.11	11.13	10.79	8.75
Count (RRSE < 100)	1	5	6	6
Average (all)	18.65	13.59	11.93	12.89

**Table 10:** Distance from LAS to OAS results.

Crop	Distance from optimal			
	SLR	MLR	M5′	ANN
PJ01	184	30	26	35
CBP02	145	7	113	1
CBA03	43	111	69	5
CBM04	232	170	6	6
CP05	96	9	23	1

Average	140	65.4	47.4	9.6

**Table 11:** Correlation coefficient between the counts of attributes in LAS-OAS intersection and RRSE.

Regression technique	*R* Correlation between RRSE and LAS-OAS intersection	*R* Correlation between RRSE and attributes left out from OAS
SLR	−0.707	0.347
MLR	−0.542	0.150
M5′	−0.641	0.034
ANN	−0.068	0.340
